# Organization of antibiotic stewardship in Europe: the way to go

**DOI:** 10.1007/s10354-020-00796-5

**Published:** 2021-02-09

**Authors:** Winfried V. Kern

**Affiliations:** Division of Infectious Diseases, Department of Medicine, University Hospital and Medical Center and Faculty of Medicine, Albert-Ludwigs-University, 79106 Freiburg, Germany

**Keywords:** Antibiotic policy, Antibiotic consumption, Antimicrobial stewardship, Antimicrobial resistance, Prescribing quality

## Abstract

It is more than two decades ago that a European Union conference on “The Microbial Threat” hosted by the Danish Government in Copenhagen in September 1998 issued recommendations to encourage good practice in the use of antimicrobial agents and reduce inappropriate prescribing. Essential components of those recommendations were antimicrobial teams in hospitals and the use of feedback to prescribers as well as educational activities. Two decades later, important surveillance systems on both antimicrobial resistance as well as on antibiotic consumption are functioning at the European level and in most European countries; European Committee on Antimicrobial Susceptibility Testing (EUCAST) has thoroughly re-evaluated, standardized and harmonized antibiotic susceptibility testing and breakpoints; there have been educational activities in many countries; and stewardship teams are now included in many guidelines and policy papers and recommendations. Yet, antimicrobial resistance problems in Europe have shifted from methicillin-resistant *Staphylococus aureus* (MRSA) to vancomycin-resistent *Enterococcus faecium* (VRE) and to multidrug-resistant gramnegative bacteria, while antibiotic consumption volumes, trends and patterns across countries do not show major and highly significant improvements. The way to go further is to recognize that better prescribing comes at a cost and requires investment in expert personnel, practice guideline drafting, and implementation aids, and, secondly, the setting of clear goals and quantitative targets for prescribing quality.

## Background

Infections due to antibiotic-resistant microorganisms are increasing in prevalence and of growing global concern. They may be associated with prolonged in-hospital stay, increased morbidity and mortality, and heightened cost [1, 2]. Antibiotic overuse and misuse are major drivers of this problem, while the development of new effective antimicrobials has become more and more limited. Between 2000 and 2015, the global antibiotic consumption rate increased by almost 40%, and a major part of the increase was in low- and middle-income countries [3]. Inappropriate antibiotic prescribing has been documented in many settings—both in human and veterinary medicine—but the patterns of misuse as well as the reasons for it differ from country to country and from region to region. Also, misuse and overuse may be prevalent in hospital medicine, primary care or both, and clearly require different approaches to control and focussed quality improvement projects.

Antibiotic stewardship in human medicine can be defined as ongoing efforts by a healthcare institution—whether local, regional/national or international—to optimize antimicrobial use among patients with the aim to improve patient outcomes, ensure cost-effective therapy and reduce adverse sequelae of antimicrobial use including antimicrobial resistance. The overall aim is to preserve the efficacy of antibiotics for use in the future. The term antibiotic stewardship was probably first coined in 1996 by John McGowan and Dale Gerding [4], mentioned shortly thereafter in the 1997 joint guideline of the Society for Healthcare Epidemiology of America (SHEA) and the Infectious Diseases Society of America (IDSA) [5], and later also used in the European context by Ian Gould [6]. On the political scene, a first position paper on antibiotic stewardship appeared as so-called Copenhagen Recommendations after a European Union conference on “The Microbial Threat” hosted by the Danish Government in Copenhagen in September 1998 [7]. The essential recommendations under the heading “Encouraging good practice in the use of antimicrobial agents” in this document were

- Educational initiatives for both health professionals (human and animal) and the general public are of major importance for improving the use of antimicrobial agents.
- Antimicrobials for therapeutic use should be prescription-only medicines and so should not be advertised to the public.
- Antimicrobial teams (including infectious disease specialists, clinical microbiologists and other appropriate specialists) should be introduced in every hospital. They should have the authority to modify antimicrobial prescriptions of individual clinicians in accordance with locally accepted guidelines, always taking account of the needs of the patient. Clinicians should be given an opportunity to approve the remit and recommendations of the teams. The teams should also cover the community, including nursing homes and other residential institutions, and the primary/secondary care interface. Feedback should be provided to clinicians.
- Guidelines for appropriate antimicrobial usage should be introduced in all aspects of both medical and veterinary practice.
- Treatment should be limited to bacterial infections, using antibiotics directed against the causative agent, given in optimal dosage, dosage intervals and length of treatment with steps taken to ensure maximum patient concordance with the treatment regimen, and only when the benefit of the treatment outweighs the individual and global risks.
- Steps must be taken to increase access to diagnostic testing for patients with infections, and the range of tests needs to be improved.

Only a few years later, the European Commission issued the Council recommendation “2002/77/EC” on the prudent use of antimicrobial agents in human medicine [8]. Surveys among the EU members regarding implementation of those recommendations followed in 2009, 2011 and 2015. Many initiatives regarding surveillance, research and education at the European and individual country level have been started since then. Stewardship as the way to promote responsible use has now also been recognized by the World Health Organization as one of the five essential global collective actions to address the problem of antimicrobial resistance (Global Action Plan on AMR [9], approved by the World Health Assembly in 2015).

## Milestones and key projects at the European level

To facilitate and further develop surveillance in the areas of antibiotic resistance and antibiotic consumption at the European level, the new European Centre for Disease Control and Prevention (ECDC, founded in 2005) was a major step. Two surveillance projects most important for antibiotic stewardship were transferred to the ECDC in the following years. One was the European Antimicrobial Resistance Surveillance System (EARSS), established in 1998 and initially funded by the European Commission’s Directorate General for Health and Consumer Affairs (DG SANCO) and the Dutch Ministry of Health, Welfare and Sports [10]. The administration and coordination of this network was transferred in early 2010 and then renamed as the European Antimicrobial Resistance Surveillance Network (EARS-Net).

The second important surveillance programme, the European Surveillance of Antimicrobial Consumption (ESAC) project, also granted by DG SANCO, was launched in 2001. ESAC, at that time coordinated by the University of Antwerp, initiated an international network of surveillance systems, aiming to collect comparable and reliable data on antibiotic use in Europe. It was transferred to the ECDC in 2011 and renamed “ESAC-Net”. The ESAC investigators published a number of most critical papers on antibiotic use density in European countries at a population-based level [10–12]. ESAC also developed and refined the methodology of point-prevalence surveys later adopted by ESAC-Net and national focal points [13–15]. ESAC-Net eventually improved the European hospital antibiotic use database which now includes data from 24 countries (compared with 30 countries in the community antibiotic use database) [16].

Other most relevant initiatives in Europe were projects associated with the European Society of Clinical Microbiology and Infectious Diseases (ESCMID), namely the European Committee on Antimicrobial Susceptibility Testing (EUCAST) (www.eucast.org), a committee that standardizes antimicrobial susceptibility testing and harmonizes breakpoints, organized by ESCMID, ECDC and national breakpoint committees, and the ESCMID study groups for Antimicrobial Stewardship (ESGAP), for Antimicrobial Resistance Surveillance (ESGARS) and for PK/PD of Anti-Infectives (EPASG).

Particularly ESGAP, founded in 1999, was extremely active in addressing issues not covered well by other bodies, such as teaching/education of medical students in antibiotic prescribing, staffing of hospital antibiotic stewardship programmes, a review of hospital antibiotic stewardship best practices, coping with controversial topics in guidelines and with access to old antibiotics [17].

There have been many more projects internationally and at country level, of course, but the above European initiatives have been and still are key to providing essential surveillance data, insight und transparency, national benchmarking options and progress in laboratory standards in the field. And they have also fostered significant research and educational activities.

## Achievements and areas for improvements

### Surveillance data do not show major improvements

Data from surveillance and research projects are prerequisites for defining areas for potential improvement and further research need and investment. What do the data tell us after two decades of surveillance? For some it may be a surprise, for others it may be as expected that the published data on antibiotic consumption and resistance continue to show wide variations in Europe [16, 18], and even within countries. Also, the patterns of resistance and antibiotic use density, admittedly, have not changed substantially over the past years, with countries like Sweden, Norway, Finland, the Netherlands and Switzerland showing limited resistance and limited antibiotic consumption, while many countries in the South show both increased resistance levels and also increased antibiotic use and/or a shift from narrow-spectrum towards broad-spectrum antibiotics. Although the rate of resistance to methicillin/oxacillin resistance among isolates causing *Staphylococcus aureus* bacteremia has decreased over the years in many countries, there were substantial increases in vancomycin resistance among *Enterococcus faecium* bloodstream isolates [19]. Also, there has been no increase in the rate of *Escherichia coli* bloodstream isolates with combined resistance to fluoroquinolones, third-generation cephalosporins and aminoglycosides in the past five years or so, but there is extreme variability in the rate of fully susceptible *Klebsiella* bloodstream infection isolates between European countries (<1% to >90%) as well as in the rate of carbapenem resistance (<1% to >50%) [18].

### Measurements of appropriate/optimized prescribing are missing

It is important to note that the “makers” of resistance surveillance systems in Europe as well as within countries in Europe (and elsewhere) are neither politicians nor prescribers themselves, and all sides may have substantial conceptual misunderstandings of the bacterial resistance problem and its consequences and solutions [20]. While surveillance systems remain important, they do not provide enough actionable information on the appropriateness and quality of prescribing, which is difficult to define and measure [21–23]. Primary care physicians often do not know which practical consequences they should draw to address a resistance problem in their region or nation. Guidance and/or adequate information may be missing. The resistance problem may be ill-defined and difficult to be identified as such for them. The situation may be slightly better for hospital medicine. Here, educational activities have been initiated in several regions and countries. A good example is the former ABS International project of colleagues in Austria and Belgium, the Czech Republic, Germany, Hungary, Italy, Poland, Slovenia and Slovakia [24, 25], which was a model for a similar education initiative across Germany and eventually also fostered the writing of an Austrian–German practice guideline on antibiotic stewardship in hospitals [26]. Examples of well-functioning systems of guidelines with surveillance and feedback may also be the Swedish Strategic Programme Against Antibiotic Resistance (www.strame.se) and the Dutch Working Party on Antibiotic Policy (Dutch acronym is SWAB, www.swab.nl).

### Better prescribing comes at a cost

There has been some work on defining and refining prescribing quality indicators, but very limited use of these [27–33]. This work includes process of care indicators that may be difficult and cumbersome to apply across healthcare systems. It also includes (more or less consented) structural indicators for quality prescribing in both outpatient and inpatient settings that do exist but are not widely applied. One of the reasons for this lack of systematic audit and quality assessment may be the increasing recognition that better care (and better prescribing) means more investment in manpower, in diagnostic facilities, in education and in stewardship activities. Only recently has the ECDC explored—through their point-prevalence surveys—the availability of an “antimicrobial stewardship consultant” (not very well defined) in hospitals in Europe, which is highly relevant regarding staffing status and need [14]. Interestingly, the median value for full-time equivalent positions across hospitals was <0.1 per 250 beds, ranging from 0 (in 16 out of 32 participating EU countries) to >0.5 per 250 beds (in Ireland and Northern Ireland only), which is considered a minimum by several institutions [34, 35].

### Quantitative targets and “smart” objectives are the way to go

At the European and also at the level of many countries in Europe, quantitative targets—for antibiotic use, for resistance containment or reduction, as well as for structural requirements and investments—are too often missing. Only 8 out of 28 European countries have defined quantitative targets in their national plans according to a survey in 2017 [36]. Among those targets were a reduction of total antibiotic consumption by 20–30% in some countries, but also more specific targets such as increases in the proportion of patients with community-acquired pneumonia not requiring intensive care initially treated with penicillin by >60% (Sweden) or the reduction by >50% of the proportion of inappropriately prescribed antibiotics across the entire healthcare chain (the Netherlands). Since then, there has been progress in setting targets. For example, Belgium published an action plan 2014–2019 with specific targets for the hospital setting that should be accomplished by 2019 including that 90% of antibiotic prescriptions should be in line with the local guidelines, that 90% compliance with local guidelines for both the choice and duration of surgical prophylaxis should be achieved, and that the indication of the prescription should be available in the medical file in 90% [37]. Examples of quantitative targets are also available for Sweden and the United Kingdom [38, 39].

The setting of such targets in the field of antibiotic stewardship after thorough discussion and consensus between clinicians, public health officials and politicians is where Europe and its countries and regions need to improve which requires specific, measurable, achievable, relevant and timebound (SMART) objectives [40].
